# Wind-blown Sand Electrification Inspired Triboelectric Energy Harvesting Based on Homogeneous Inorganic Materials Contact: A Theoretical Study and Prediction

**DOI:** 10.1038/srep19912

**Published:** 2016-01-28

**Authors:** Wenwen Hu, Weiwei Wu, Hao-miao Zhou

**Affiliations:** 1 College of Information Engineering, China Jiliang University, Hangzhou, 310018, PRC; 2 College of Materials and Textiles, Key Laboratory of Advanced Textile Materials and Manufacturing Technology of the Ministry of Education, Zhejiang Sci-Tech University, 310018, PRC; 3 Department of Chemical Engineering & Russell Berrie Nanotechnology Institute, Technion – Israel Institute of Technology, Haifa, 3200003, Israel

## Abstract

Triboelectric nanogenerator (TENG) based on contact electrification between heterogeneous materials has been widely studied. Inspired from wind-blown sand electrification, we design a novel kind of TENG based on size dependent electrification using homogeneous inorganic materials. Based on the asymmetric contact theory between homogeneous material surfaces, a calculation of surface charge density has been carried out. Furthermore, the theoretical output of homogeneous material based TENG has been simulated. Therefore, this work may pave the way of fabricating TENG without the limitation of static sequence.

In the earth’s crust, silicon is the second most abundant element and various forms of silica is the most common composition unit of sand. For wind-blown sand granular systems, such as dust devil, sand storm, wind-blown sand saltation, etc, the upward electric field phenomenon originated from the positively charged large particles saltate/creep near the surface and small negatively charged smaller particles suspend into the air is widely observed^1^. The charged wind-blown sand granular systems always cause various damages, especially to the attenuation of electromagnetic wave signals caused by the light scattering and absorption of wind-blown sand&dust^2^. Nowadays, people reluctantly seem this huge static electric energy in those systems as harmful and waste energy and suffers their damages. However, an interesting and desired topic, how to take advantage of this natural phenomenon and convert the tremendous, harmful energy to useful electricity, hasn’t been paid enough attention and is a lack of study.

Based on different physical rules including electromagnetic induction effect^3^, electrostatic effect^4^, piezoelectric effect^5^ and so on, human beings convert mechanical energy to electricity that is one of the main kind of power source. Since 2012, triboelectric nanogenerator (TENG) has been invented as a novel technology to harvest waste mechanical energy which is a possible solution of not only portable electronics but also the energy crisis for human beings^6^. Comparing with piezoelectric materials based nanogenerator, TENG represents some unique merits such as high conversion efficiency, low fabrication cost, reliable robustness, which attaches great research interests both on theoretical and experimental studies^7^,^8^,^9^,^10^,^11^,^12^,^13^.

Generally, the working principle of TENG includes two processes^9^,^14^. First, two different kinds of materials located at different sites of electrostatic sequence obtain opposite charges on the surface via contact electrification with or without rubbing. Second, the electrodes made of oppositely charged materials move to change the capacitance for creating an electric potential difference that drives the free electrons in the external circuit to flow back and forth as alternating current (AC). Both in contact and sliding mode, the electrification between two different materials is a key step for generating electricity^12^,^15^,^16^. Till now, TENGs have been developed by assembling two different kinds of materials with the empirical arrangement in the static sequence, for example, PET and Kapton^6^, PTFE and Al^17^, PDMS and Al^18^, PMMA and Kapton^19^, PDMS and ITO^20^, TiO_2_ and PTFE^21^, PDMS and Au^22^, PVDF and nylon^23^, etc. With the limitation of static sequence, almost either of these two kinds of materials is polymer. Contact electrification occurs not only between heterogeneous but also homogeneous materials, which has been demonstrated by several experimental works, such as silica-silica^24^,^25^,^26^,^27^,^28^,^29^, aluminum oxide-aluminum oxide^25^, polymer-polymer^30^,^31^,^32^. Some homogeneous materials based TENGs also have promising performance^33^. Although polymer is always cheap, flexible and light, polymer based TENG is highly unfavorable to work in harsh environment such as desert, outer space and so on for its narrow working temperature area, fast aging and poor antiwear property^34^. Therefore, it is valuable to design and develop a new kind of TENG made with total inorganic materials like silica-silica to work in special environment.

For the electrification between identical materials, high energy trapped surface states theory proposed by Lowell and Truscutt^35^ has been widely used to explain charge transfer during asymmetric rubbing^26^,^36^,^37^ Based on high energy trapped states theory, particle-size-dependent charging, such as larger particles tends to be positively charged and smaller particles tends to be negatively charged, has been well explained^26^,^28^,^36^,^37^,^38^. In the wind-blown sand granular system, mechanisms of size-dependent electrification between identical insulator particles have been widely studied^16^,^26^,^36^,^37^,^38^,^39^,^40^, such as asymmetric contact between two particles with transfer of high-energy trapped electrons^37^ or holes^26^ (HETH). In our previous work, a contact charge model of high-energy trapped holes has been developed^26^ and verified with experiments^24^,^26^ for collision of homogeneous silica particles to predict the size-dependent contact electrification. From the experimental point view, silica film, made with earth abundant element oxygen and silicon, is chemical inertia and compatible with many semiconductor fabrication process like thermal oxidation^41^, magnetron sputtering^42^, sol-gel coating^43^, plasma enhanced chemical vapor deposition^44^, atom layer deposition^45^ and so on. Meanwhile, the synthesis of silica nanoparticles is also widely studied for many years^46^. Therefore, inspired by these natural phenomenon and studies, using homogenous silica based materials with different sizes as the contact electrification layer to fabricate TENG for energy harvesting, is highly feasible, which may pave a new way to develop a novel kind of TENG working in harsh environment.

In this manuscript, we propose a new kind of TENG based on contact electrification between homogeneous silica materials with different sizes for harvesting energy. A comprehensive theoretical model is established to understand the effect of size dependent elasticity on the surface charge density of chemically identical silica nanoparticles under normal loads. To our best knowledge, it is the first time of calculating the surface charge density after single time contact electrification for TENG through theoretical simulation. Then, these results are applied to predict the output characteristics of the contact-mode and sliding-mode TENGs. We demonstrate that the maximum output power of TENG using earth abundant and identical silica as contact electrification layer will reach 12.98 μW and 13.92 nW in contact and sliding model respectively. Indeed, the output will be greatly enhanced after multi-times contact electrification for more accumulated charges and higher surface charge density^30^,^47^ Furthermore, the whole TENG is covered with homogenous materials (silica), which may broaden the application of TENG in harsh environment.

## Results

**Figure 1:**
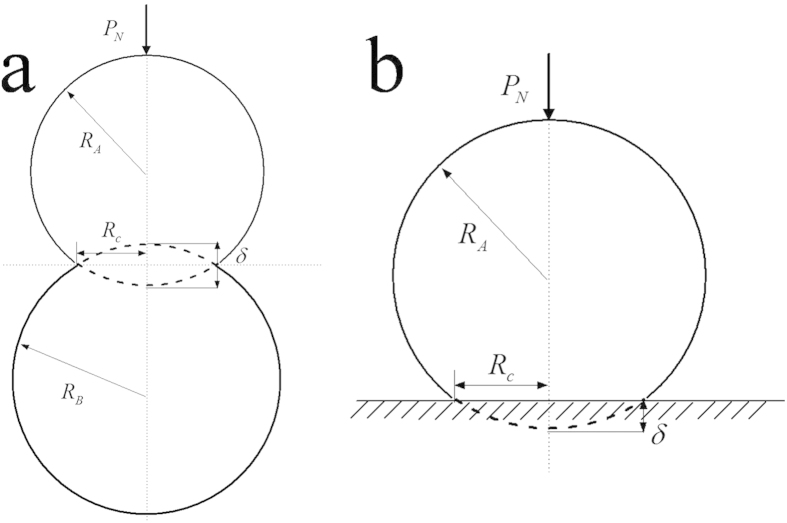
Scheme of the contact of a sphere with radius *R*_*A*_ on (**a**) another sphere with radius 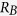; (**b**) a rigid flat object under a normal point load 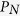. 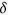 is the overlapped deformation, and 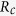 is the contact radius.

Denote two neutrally-charged sphere particles, 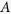 and 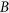 with radii 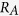 and 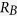 respectively, in a normally elastic colliding process, as shown in Fig. 1(a). Based on high-energy trapped hole contact charging model^26^, the net charge transfer of particle 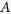
equals to the number of high-energy trapped holes gained from particle 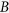 to particle 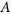 subtracting that lost from particle 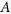 to particle 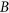, that is,


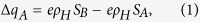


where 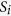 is the surface area of the contact part of sphere particle 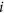 (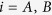), 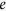 is the elementary charge, 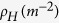 is the surface density of the high-energy trapped holes which is assumed to be identical for all particles initially^26^,^37^ The net charge transfer 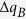 of particle 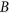 equals to 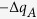.When 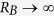, Hertzian normal contact of two spheres will become the contact between a sphere and a rigid flat, as shown in Fig. 1(b). If the contact surface radius 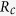
is given, the surface area of the contact part 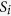 of sphere 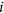 can be predicted,


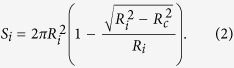


The net charge transfer of normal contact spheres is determined by the difference of surface areas of contact part 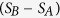. Let 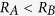, the contacted surface area 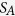 will be greater than the contacted surface area 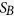, and then the net charge transfer 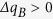 and 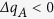. If pre-collisional particles are both neutrally charged, the larger particle tends to be positively charged and the smaller particle tends to be negatively charged.

**Figure 2:**
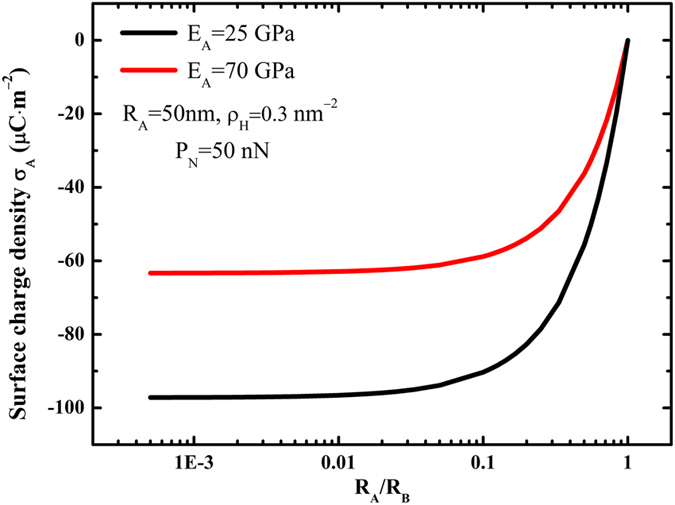
For Hertzian contact of two spheres under normal load *P*_*N*_ = 50 nN, the surface charge density 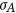 of sphere *R*_*A*_ varies with the radii ratio *R*_*A*_/*R*_*B*_.

In order to achieve an optimal design of TENGs, the surface charge density is a vital parameter determining the output characteristics. When 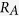 is fixed, Fig. 2 shows that the surface charge density 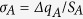 of the post-collisional particle 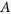 decreases with radius 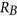 (or increases with 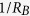) and finally reaches its minimum value while 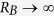 (or 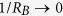). When the radius 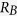 tends to infinite, the particle-particle Hertzian contact can be treated as the particle-plane contact, and in such case the surface charge density tends to its extreme value. Therefore the particle-plane Hertzian contact case will be an optimal design to obtain the maximum charge & power outputs for TENGs.

**Figure 3:**
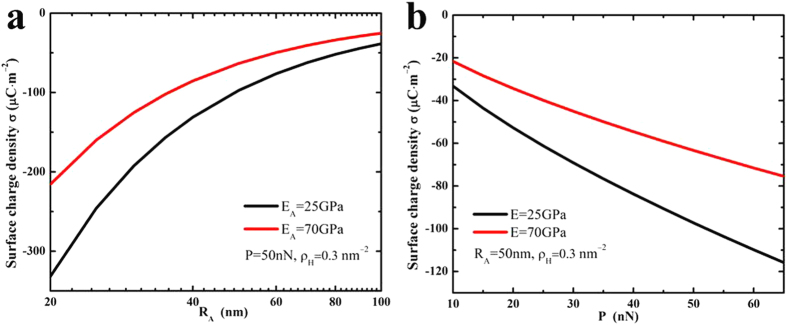
For Hertzian contact between a sphere and a plate, the surface charge density 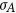 varies with (**a**) radius R_A_ under normal point load 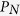 = 50 nN; and (**b**) normal point load P with 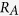 = 50 nm.

In the case of elastic particle-plane Hertzian contact, we predict the surface charge density 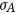 varying with the normal load 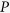 and the radius 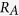, as shown in Fig. 3. The absolute value of the surface charge density, 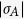, increases with the normal load 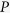 increasing, but decreases with the increase of the radius 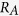.

Materials reduced to submicron or/and nanoscale show different mechanical properties compared to what they show on macroscale. Wang *et al*.^48^ pointed out that the Young’s module of silica nanowires of 100 nm diameter SiO_2_ nanowires is much lower than that of bulk SiO_2_ materials. Since the Young’s module reveals the strain-stress relationship, it will have an impact on the contact area of Hertzian contact and also on the contact charge. Figures 2 and 3 demonstrate that the Young’s module have significant effects on the surface charge density. For instance, as shown in Fig. 2, when 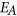 = 25 GPa, the absolute value of the extreme surface charge density, will be 53.5% greater than the case 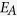 = 70 GPa, respectively. Therefore, in this study we consider the nanoscale effect and take the Young’s module as 25 GPa.

**Figure 4:**
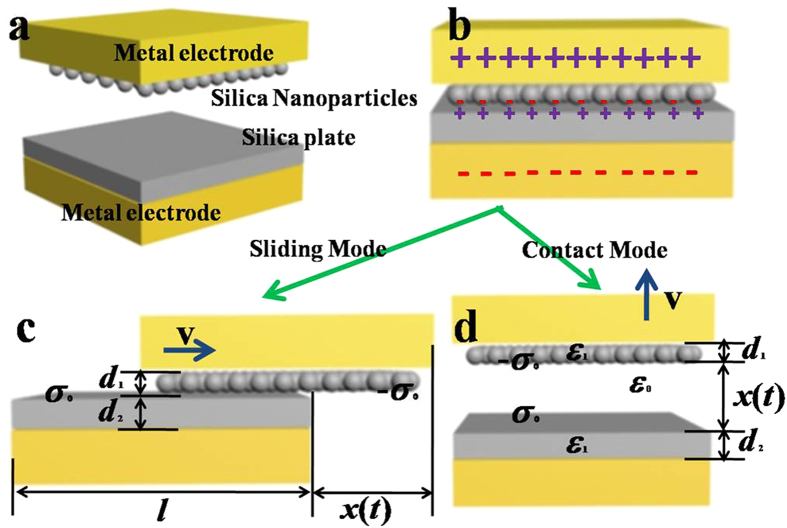
Scheme of (**a**) contact-mode and (**b**) sliding-mode TENGs. The top surface is decorated with SiO_2_ nanoparticles, and the bottom surface is flat SiO_2_ plate. After separation, the bottom surface has the surface charge density 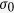 and the top surface the surface charge density 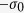.

Instead of taking advantage of contact electrification between different insulators^6^,^17^,^18^,^19^,^20^,^21^,^22^,^23^, we choose the identical silica insulators with the property of size-dependent polarity during contact electrification. Figure 4 shows the schemes of contact-mode and sliding-mode TENGs. The top surface is decorated with a layer of SiO_2_ nanoparticles, and the bottom surface is flat SiO_2_ plate. Thin layers of metal film are deposited on two SiO_2_ material plates as the metal electrodes. The metal electrodes in the upper and lower plates are called the top and down electrodes, respectively.

Figure 4(a) is the schematic diagram of the structure of our TENG containing two metal electrodes, silica nanoparticle and silica plate. Initially, the two surfaces are neutrally charged. As observed in wind-blown sand granular systems and our previous work, the surface coated with SiO_2_ nanoparticles will be negatively charged and the SiO_2_ plane surface will be positively charged after single time contacting^26^, and the bottom surface has the surface charge density 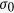 and the top surface the surface charge density 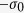
as shown in Fig. 4(b–d) are the schematic diagrams of sliding and contact mode separately. After separation, the electric charge of a single SiO_2_ nanoparticle will be


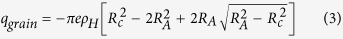


If there are N particles decorated on a plane with the surface area 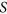
(*S* = *wl*, 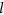: length; 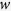: width), the number density will be 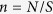. After separation of two planes, the total electric charge of nanoparticle-decorated top plane will be,


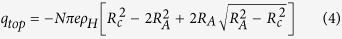


And the averaged surface charge density of the down plane 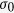 will be


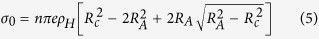


**Figure 5:**
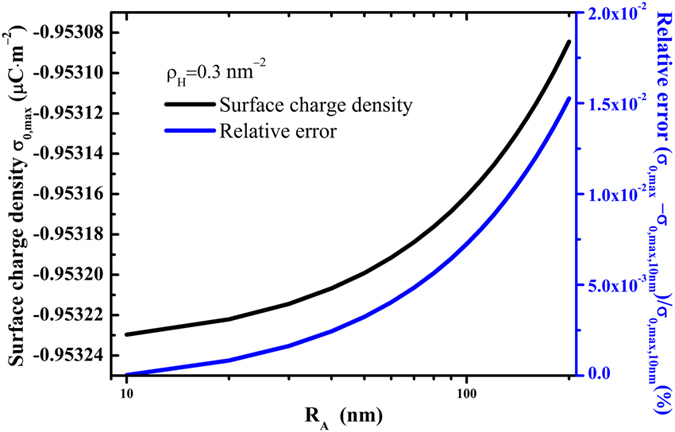
For a square surface fully decorated with nanoparticles, the maximum surface charge density 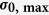 varies with the radius *R*_*A*_.

Then, the averaged surface charge density of the top plane will be 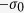. For a rectangle plane with length 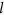 and width 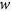, the maximum number of particles coated on such surface will be 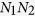, where 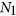 and 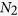 can be solved by 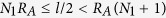 and 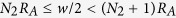, respectively. Noticeably, the surface charge density 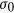 increases with 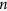, and the maximum surface charge density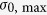 will be 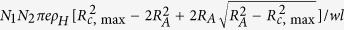, where 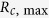 denotes the maximum elastic contact surface radius which can be solved according to the critical normal load. For a square surface, 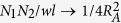, and the maximum surface charge density 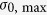
tends to be 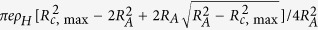. For instance, when 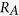 = 10 nm,the maximum surface charge density. Figure 5 shows that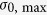 slightly increase linearly with 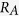 and is nearly a constant value −0.953 μC m^−2^ with 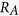 in the range 10 ~ 100 nm, and the relative error 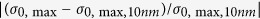 is very small. The above maximum surface charge density means that particles are densely distributed, and this is an ideal state. While in this ideal state, the squeezing action between a particle and its adjacent particles under external forces also affects the Hertzian deformation of particle-plane contact. In order to avoid such inter-particle effects, sparse distribution of nanoparticles is more reasonable. For example, when a quarter of the maximum number of particles such as 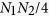 are decorated on the top surface, the surface charge density will be 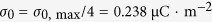.

**Figure 6:**
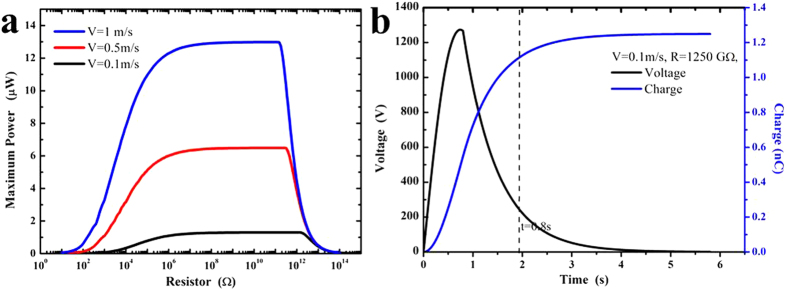
Outputs of contact-mode TENGs with uniform velocity of separation. (**a**) Maximum power outputs vary with resistance; (**b**) Under the optimum power output (R = 1250 GΩ, v = 0.1 m/s), voltage & charge output profiles change with time.

**Figure 7:**
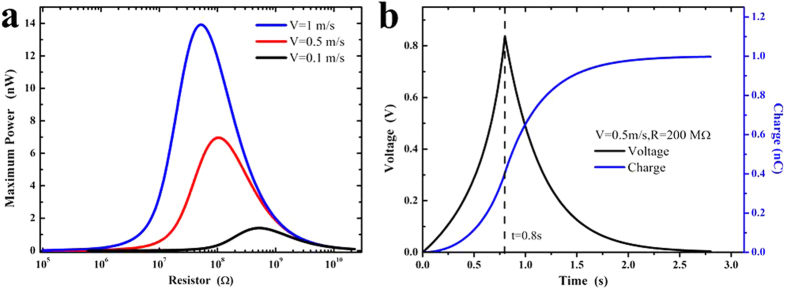
Outputs of sliding-mode TENGs with uniform velocity of separation. (**a**) Maximum power outputs vary with resistance; (**b**) Under the optimum power output (R = 200 MΩ, v = 0.5 m/s), voltage & charge output profiles change with time.

For normal contact-mode and sliding-mode TENGs connected to an arbitrary resistor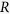, the output properties have been respectively discussed^14^,^49^. Figures 6 and 7 respectively depicts the theoretical output properties of normal contact-model and sliding-mode of TENGs. The parameters of our TENGs are given in Table 1. For instance, the width 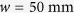, the length *l* = 100 mm, the thicknesses 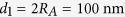, and 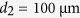 for both contact-mode and sliding-mode TENGs. The maximum departing distance of contact-mode TENGs is 80mm, and the maximum sliding distance 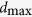 of sliding-mode TENGs is also 80mm. When the velocities are respectively 0.1 m/s, 0.5 m/s, 1 m/s, theoretical calculations of maximum power outputs are carried out with different resistances, as shown in Figs 6(a) and 7(a). Obviously, there will be optimum values of resistance 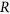 for contact model and sliding-mode^14^,^49^. For instance, as shown in Fig. 6(a), when the power outputs reach their optimum values 1.298 μW, 6.491 μW and 12.98 μW after single time contact electrification for contact-model TENGs, the resistances are respectively 1250 GΩ (separating velocity is 0.1 m/s), 275 GΩ (separating velocity is 0.5m/s), and 125 GΩ (separating velocity is 1 m/s). For sliding-model TENGs, the maximum power output could reach 13.92 nW after single time electrification when sliding velocity is 1 m/s. According to optimization of resistance value, Figs 6(b) and 7(b) respectively demonstrate the voltage and charge outputs of contact-model TENGs and sliding-mode TENGs when separating & sliding velocities are both 0.1m/s. Therefore, our optimized designs of contact-mode and sliding-mode TENGs based on contact electrification of homogeneous materials with different sizes would provide a new way for harvesting natural wind-blown energy to drive the nano/micro scale instruments.

## Discussion

Inspired from wind-blown sand electrification, we design a novel kind of TENG based on size dependent electrification using identical silica materials for harvesting energy. A theoretical mode is established to elucidate the mechanism of the contact electrification process and a calculation of surface charge density has been carried out. Furthermore, the output of homogeneous material based TENG has been simulated. We demonstrate that the maximum power outputs of TENG using earth abundant and homogenous silica as contact electrification layer will reach 12.98 μW and 13.92 nW in contact and sliding model after single time contact electrification, respectively. Indeed, the output will be greatly enhanced after multi-times contact electrification for more accumulated charges and higher surface charge density. The design of our TENG is polymer free and breaks the limitation of static sequence, which may broaden the application of TENG in harsh environment.

## Method

The high-energy trapped hole contact charging model^26^ is used to predict the net charge transfer and the surface charge density when identical SiO_2_ particle-particle or particle-plane contact. The net charge transfer is related to the Hertzian contact area.

According to Hertzian normal contact theory, each sphere will undergo a normal deflection and a contact surface when the two spheres are subjected to a normal load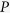, as shown in Fig. 1(a). And the normal deflection 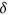 is given by,


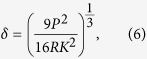


where *K* is a material constant and commonly referred to as the effective stiffness,


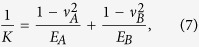


where 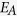, 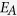 are the Young’s modules and 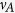, 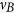 are the Poisson’s ratios, respectively. The effective radius of curvature 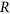 of the two spheres is defined as,


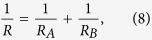


where 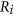 is the radius of sphere 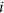 (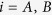).When 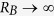, Hertzian normal contact of two spheres will become the contact of a sphere and a rigid flat, as shown in Fig. 1(b). The contact surface radius 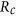 and the normal deflection 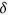 satisfy 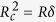, and thus the contact surface radius 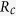 will be,


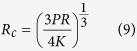


Here, we only consider the elastic Hertzian contact; therefore we need to calculate the critical normal load 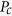 from elastic stage to plastic stage. Love *et al*. demonstrated the stress produced in a semi-infinite body^50^,


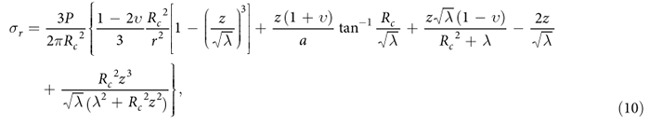



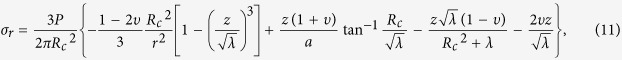



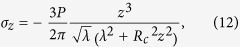



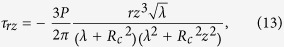


where 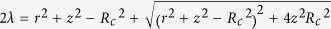. Under normal point load, the plastic deformation starts at the contact area, and the initially yielding point will be on the axis of 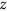 of the elastic semi-infinite body. When 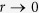, the stress at points on the axis of 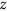 will be


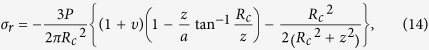



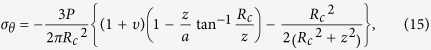



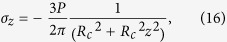



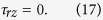


Here, 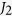, the second invariant of stress deviator tensor is applied to predicted the critical normal load, and 
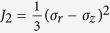
. According to the Von Mises criteria, the yielding function 
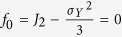
 for axial extension experiments, where 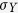 is the yield strength. Therefore, the critical normal point load can be derived by solving function 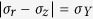.

Finally, we carry out a theoretical study and prediction of wind-blown sand electrification inspired triboelectric energy harvesting based on homogeneous inorganic materials contact. For normal contact-mode and sliding-mode TENGs connected to an arbitrary resistor 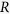, the charges on the top electrode as a function of time are respectively given as^14^,^49^,


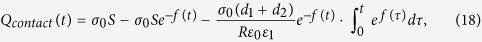



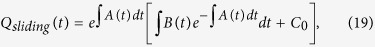


where 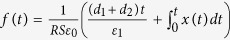, 
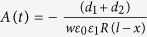
, 
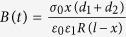
. The current, voltage and power outputs are respectively,


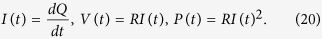


## Additional Information

**How to cite this article**: Hu, W. *et al*. Wind-blown Sand Electrification Inspired Triboelectric Energy Harvesting Based on Homogeneous Inorganic Materials Contact: A Theoretical Study and Prediction. *Sci. Rep*. **6**, 19912; doi: 10.1038/srep19912 (2016).

## Figures and Tables

**Table 1:** Parameters.

Radius of SiO2 particle	R_A_ = 50 nm
Dielectric of SiO_2_	*ε*_*1*_ = 3.9, *d*_*1*_ = 100 nm, *d*_*2*_ = 100μm
Width & Length	*w* = 50mm, *l* = 100mm,
Velocity	*v* = 0.1, 0.5, 1 m/s
Maximum distance	*x*_*max*_ = 80mm, *d*_*max*_ = 80 mm
Poison ratio	*ν*_*A*_ = *ν*_*B*_ = 0.3
Young’s module	*E*_*A*_ = 25 GPa, *E*_*B*_ = 70GPa
Yield strength	σ_Y_ = 80 MPa
Surface density of HETHs	ρ_H_ = 0.3 nm^−2^
